# Purinergic transmission in brain tumors and its impact on drug development

**DOI:** 10.1186/2193-1801-4-S1-L10

**Published:** 2015-06-12

**Authors:** Maria Abbracchio, Stefania Ceruti, Davide Lecca

**Affiliations:** Dept Pharmacol Sci, University of Milan, Italy

**Keywords:** Cell differentiation, purinoceptors, new targets in glioma

Extracellular nucleoside and nucleotides acting via adenosine/P1 and nucleotide P2 (P2X/P2Y) purinoceptors are fundamental signaling molecules controlling the survival and proliferation of astrocytes and oligodendrocytes (Ceruti & Abbracchio, Adv Exp Med Biol 986:13, 2013). The malignant transformation of these cells to progressively more aggressive tumors (respectively, astrocytomas and anaplastic glioblastoma, and glioblastoma multiforme, containing proliferating cells resembling Oligodendrocytes Precursor Cells, OPCs) confers growth advantage and chemoresistance. Characterization of the specific P1 and P2 receptors on these tumors may unveil new strategies to reduce cancer growth and/or promote differentiation to non-cancerous glial phenotypes. The adenosine A3 receptor (A3AR) has emerged as a potential target. Under hypoxia, a condition typical of gliomas’ core, A3AR mediates chemoresistance via the PKB/Akt pathway (leading to inactivation of the pro-apopototic Bad protein) and by upregulating matrix metalloproteinase-9, that degrades extracellular matrix and promotes migration of glioma cells towards healthy brain regions (Ceruti & Abbracchio and ref therein). Thus, inhibition of A3AR with selective antagonists could represent an appealing therapeutic approach. More recently, the P2X7 receptor has been recently found to be over-expressed in grade IV human gliomas (Monif et al., J Inflammation, 11:25, 2014) and its blockade with the synthetic antagonist Brilliant Blue G decreased tumour cell number. Finally, treatment of human glioblastoma multiforme cells with UDP, UDP-glucose or LTD4, that act as agonists at the new P2Y-like GPR17 receptor, reduced the formation of glioma spheres, suggesting that GPR17 stimulation on highly proliferating tumor OPCs may drive their differentiation to the oligodendroglial fate, negatively affecting both tumor proliferation and self-renewal (Dougherty et al., Cancer Res 72:4856, 2012).

